# Supplementary material for: Content-rich biological network constructed by mining PubMed abstracts
Source: BMC Bioinformatics. 2004 Oct 8;5:147. doi: 10.1186/1471-2105-5-147 (PMC528731; doi:10.1186/1471-2105-5-147)
Supplement: Additional File 5 — The original Chilibot query results of the term "long-term potentiation (LTP)" and 22 other terms, limiting the latest references analyzed to the years 1990, 1995, 2000, and 2004. [file 1471-2105-5-147-S5.bz2 › chilibotAdditionalFile5/ltp2000/html/LTP_ZIF268.html]

 


 **LTP** and **ZIF268** 
  
Found 9 abstracts in PubMed,  **9 abstracts were retrieved and analyzed**.  


---

 Search Google  |
 PDF files only 
|  EDU domain only 

---

**Interactive relationship** (e.g. stimulation, inhibition, etc)

- The extent of  **LTP**  saturation in fascia dentata produced by the standard chronic  **LTP**  stimulation protocol was assessed both electrophysiologically and through the use of an anatomical marker activation of the immediate early gene  **zif268** .  Ref: 7931545 J Neurosci, 1994
- We have compared changes in mRNA of three genes,  **zif268** , raf B, and syntaxin 1 B, following the unilateral induction of long term potentiation  [ **LTP** ]   **LTP**  in rats previously trained in a water maze, and in behaviourally naive animals.  Ref: 9714460 Neurosci Lett, 1998
- These results suggest that  **LTP**  and spatial training engage a common pathway utilizing an increase in mRNA for raf B, and demonstrate a dissociation between  **LTP**  and spatial learning with respect to expression of  **zif268**  and syntaxin 1B.  Ref: 9714460 Neurosci Lett, 1998
- Three of these genes were known only from expressed sequence tags RM1 3, two were known genes whose up regulation by  **LTP**  has not been described GADD153 CHOP and ler5, and five were known genes whose up regulation by  **LTP**  has already been reported MAPK phosphatase, NGFI A  **zif268** , vesl 1S homer 1a, Ag2, and krox 20.  Ref: 10820183 J Neurochem, 2000

**Parallel relationship** (e.g. studied together, co-existance, homology, etc.)

- In contrast, administration of maximal electroconvulsive shock led to robust  **zif268**  activation throughout the hippocampus, enhancement of synaptic responses, occlusion of  **LTP**  produced by discrete high frequency stimulation, and spatial learning deficits in the water task.  Ref: 7931545 J Neurosci, 1994
